# Spatio-temporal variations and factors of a provincial PM_2.5_ pollution in eastern China during 2013–2017 by geostatistics

**DOI:** 10.1038/s41598-019-40426-8

**Published:** 2019-03-05

**Authors:** Xue Sun, Xiao-San Luo, Jiangbing Xu, Zhen Zhao, Yan Chen, Lichun Wu, Qi Chen, Dan Zhang

**Affiliations:** International Center for Ecology, Meteorology, and Environment, Collaborative Innovation Center of Atmospheric Environment and Equipment Technology (AEET), School of Applied Meteorology, Nanjing University of Information Science & Technology, Nanjing, 210044 China

## Abstract

Fine particulate matter (PM_2.5_) is a typical air pollutant and has adverse health effects across the world, especially in the rapidly developing China due to significant air pollution. The PM_2.5_ pollution varies with time and space, and is dominated by the locations owing to the differences in geographical conditions including topography and meteorology, the land use and the characteristics of urbanization and industrialization, all of which control the pollution formation by influencing the various sources and transport of PM_2.5_. To characterize these parameters and mechanisms, the 5-year PM_2.5_ pollution patterns of Jiangsu province in eastern China with high-resolution was investigated. The Kriging interpolation method of geostatistical analysis (GIS) and the HYbrid Single-Particle Lagrangian Integrated Trajectory (HYSPLIT) model were conducted to study the spatial and temporal distribution of air pollution at 110 sites from national air quality monitoring network covering 13 cities. The PM_2.5_ pollution of the studied region was obvious, although the annual average concentration decreased from previous 72 to recent 50 μg m^−3^. Evident temporal variations showed high PM_2.5_ level in winter and low in summer. Spatially, PM_2.5_ level was higher in northern (inland, heavy industry) than that in eastern (costal, plain) regions. Industrial sources contributed highest to the air pollution. Backward trajectory clustering and potential source contribution factor (PSCF) analysis indicated that the typical monsoon climate played an important role in the aerosol transport. In summer, the air mass in Jiangsu was mainly affected by the updraft from near region, which accounted for about 60% of the total number of trajectories, while in winter, the long-distance transport from the northwest had a significant impact on air pollution.

## Introduction

Air pollution is a worldwide environmental issue which can lead to significant ecological and environmental effects and threaten human health, especially the atmospheric fine particulate matters (PM_2.5_) in many Asian cities^1–4^. Typically in China, associated with the rapid industrialization and urbanization and massive energy consumption, air quality was deteriorated^5,6^ and haze pollution has frequently occurred^7,8^. In 2016, 75.1% cities in China exceeded the annual ambient air quality guidelines, and PM_2.5_ was the primary pollutant during most pollution days^9^. Specifically, the population-weighted mean PM_2.5_ in Chinese cities was 61 μg m^−3^, three times higher than the global mean^10^. Therefore, air pollution control polices and measures have also been gradually emphasized and strengthened, for which understanding the spatial and temporal distribution of air pollutants and related sources was the key step^11–13^. However, the PM_2.5_ distribution was significantly influenced by local terrain features, meteorological conditions, city characteristics and economic levels, local emission sources and regional pollution transport^14–18^. For instance, the PM_2.5_ concentrations in 20 monitoring sites of California, USA change daily and such change was cyclical and changed with the season in the corresponding cycle^19^. Therefore, it is desired to study the large-scale and long-term spatio-temporal distribution of PM_2.5_ and corresponding mechanisms. Nowadays, there were some high-resolution air pollution estimation methods, including interpolation method^20^ and the satellite top-down approach based on ground-based fixed-site air pollution monitoring networks^21–23^.

In this study, the 5-year high-resolution distributions of PM_2.5_ concentrations in a province with 13 cities in eastern China were investigated with air pollution sources and land uses by Geographic Information System (GIS) and backward trajectory clustering analysis. The main objectives were: (1) to explore the spatial and temporal distribution characteristics of PM_2.5_ in different geographical areas and under varied environmental managements; and (2) to illuminate the influence mechanisms of air pollution source, economic, topographic, and meteorological factors on PM_2.5_ pollution patterns.

## Results and Discussions

### Spatio-temporal distributions of the 5-year provincial air PM_2.5_ pollution

#### Intra-annual and inter-annual variations of PM_2.5_ in the overall province

**Figure 1:**
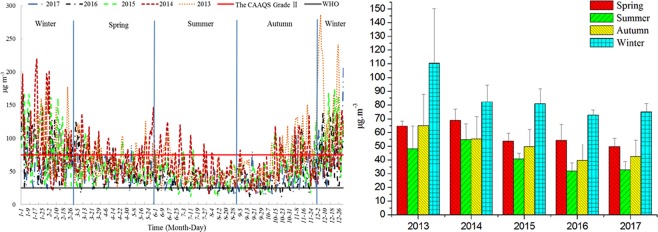
Daily and annual variations of PM_2.5_ in Jiangsu province, China from 2013 to 2017.

**Figure 2:**
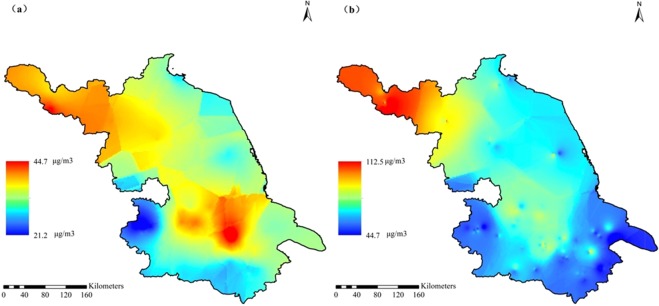
Spatial distribution of PM_2.5_ concentrations in summer (**a**) and winter (**b**) of 2017 for Jiangsu province.

The large-scale and long-term PM_2.5_ pollution was significant in Jiangsu. Compared with the Chinese Ambient Air Quality Standards (CAAQS) (Table A1), the average annual PM_2.5_ concentration of each city during 2013–2017 exceeded the Grade II guideline (Fig. A1). Intra-annually, the PM_2.5_ levels showed significant seasonal variations (Table 1), which was a U-shaped pattern of high in winter and low in summer^24^ (Fig. 1). According to the proportions of days with different PM_2.5_ levels (Fig. A2e), there were 57 days in 2017 exceeded the national Grade II standard, up to 42 days of which were in winter. The PM_2.5_ concentrations in winter and summer of 2017 were 72.5 and 32.3 μg m^−3^, respectively (Fig. 2). These seasonal phenomena were attributed to pollution sources and meteorological conditions. In winter, anthropogenic emissions related to heating demand were increased, and a combination of persistent temperature inversions and low mixed boundary layer was unfavorable to the atmospheric pollutant dispersion^25–28^. While in summer, influenced by the monsoon climate, the precipitation was high and could significantly reduce the particulates in atmosphere, and the prevailing east wind and the clean air from ocean has also dilution effects on the air pollution in Jiangsu province.

**Table 1:** Significant differences of PM_2.5_ levels among different seasons in 2013–2017.

Season	Average (μg.m^−3^)	Significance			
		Spring	Summer	Autumn	Winter
Spring	58.2	1.000	—	—	—
Summer	41.6	**0.048** ^*^	1.000	—	—
Autumn	50.2	0.320	0.282	1.000	—
Winter	88.7	0.001**	**0.000** ^**^	**0.000** ^**^	1.000

*means *P* < 0.05, **means *P* < 0.01.

**Figure 3:**
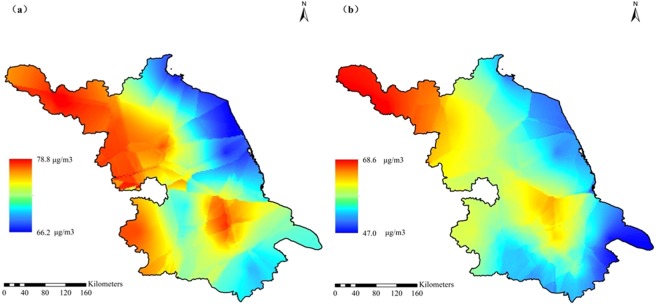
Differences of annual PM_2.5_ concentrations between 2013 (**a**) and 2017 (**b**) in the provincial distribution.

Inter-annually, the average annual concentration of the overall Jiangsu province has decreased significantly from 2013 to 2017 (Fig. 1), which was 71.8, 66.3, 57.7, 50.3, and 49.6 μg m^−3^, respectively. The proportion of pollution days has decreased simultaneously, that was 34.2%, 29.5%, 22%, 17.1%, and 15.6%, respectively. These air quality improvements were closely related to the environmental protection measures taken by the provincial government in recent years. It can be seen from Fig. 3 that the pollutants in various regions of Jiangsu Province have been reduced to varying degrees from 2013 to 2017, indicating that the government’s environmental protection measures were effective. As a key area for environmental governance, the pollution concentration of provincial capital Nanjing decreased significantly, while the air quality improvement in the northern Jiangsu was slight, which mainly due to the local heavy industry.

#### Spatial distribution of the provincial PM_2.5_

**Figure 4:**
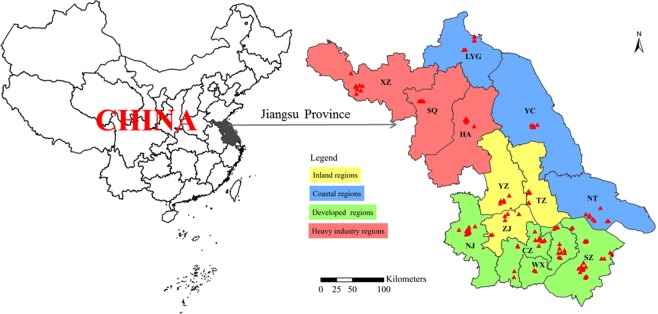
Locations of Jiangsu province in China and the 110 scattered monitored sites (solid triangles) covering all 13 cities.

Using the average PM_2.5_ values at all 110 monitoring sites in 2013 and 2017, the Kriging interpolation analysis was performed to obtain the provincial simulated distribution map of PM_2.5_ concentrations (Fig. 3). In the whole studied area, the PM_2.5_ levels decreased gradually from west to east. According to the geographical location, urbanization level, population density, civilian car ownership, total GDP and per capita GDP, all cities of this province can be divided into 4 regions (Fig. 4), including western inland, eastern coastal, northern heavy industry and south developed regions. The industrial enterprises of XZ (Xuzhou) and HA (Huai’an) were dominated by heavy industries including mining, so they were divided into northern heavy industrial area. The specific features of each city with PM_2.5_ concentrations were showed in Table 2. The PM_2.5_ level was in the order of heavy industrial > inland > developed > coastal regions. It showed a pattern that the PM_2.5_ pollution in densely populated and developed areas with more emission sources was higher than those with better vegetation coverage and self-purification capacity^28,29^. Besides the effects of urbanization, the industry and geographic location also showed significant impacts. Figure 2 showed that the pollution level in developed areas was lower than that in the underdeveloped northern regions with heavy industrial pollution emissions. Impacted by the ocean atmospheric transmission, the pollution in coastal areas with stronger self-purification capabilities was lower than that in the inland regions.

**Table 2:** Regional divisions of Jiangsu province and the corresponding PM_2.5_ concentrations in 2017.

Characteristic regions	Urbanization (%)	Density of Population (per km^2^)	Civil Vehicles Owned (ten thousands)	Total GDP (0.1 billion yuan)	Per capita GDP (thousand yuan)	PM_2.5_ (μg/m^3^)
**Heavy industry**	**60**	**629**	**65**	**3736**	**59**	**59**
XZ	62	783	102	5809	67	68
SQ	58	595	49	2351	48	57
HA	60	508	45	3048	63	51
**Inland regions**	**66**	**745**	**58**	**4128**	**103**	**54**
YZ	64	708	64	4449	99	54
TZ	63	831	62	4102	88	52
ZJ	69	697	49	3834	121	56
**Developed regions**	**76**	**905**	**201**	**10241**	**134**	**44**
SZ	76	781	313	15475	146	43
WX	76	1111	160	9210	141	45
CZ	71	782	110	5774	123	49
NJ	82	946	222	10503	127	41
**Coastal regions**	**62**	**715**	**86**	**4574**	**70**	**43**
YC	62	534	76	4576	63	44
LYG	60	626	48	2377	53	45
NT	64	986	135	6768	93	40

Because the economic development between southern and northern regions of Jiangsu province was seriously unbalanced (GDP in Table 2), to balance the huge gap, developments of heavy industry in the northern region led by machinery, electricity, chemistry, building materials and energy were vigorously promoted by governments. For instance, of the 221 key enterprises with exhaust emissions in the 13 cities of Jiangsu Province in 2018, there were up to 30 in a north heavy industry city (XZ). Moreover, in order to accelerate the process of urbanization in the northern region, the municipal construction was in the peak period, corresponding dusts were substantial and aggravated air quality.

Of course, compared with the satellite-based top-down approach based ground fixed-site air pollution monitoring networks, Kriging interpolation method has limitations. Remote sensing provides the detailed information in space and time not only from accessible areas but also from inaccessible areas^30^. Satellite-retrieved aerosol optical depth has been increasingly utilized for the mapping of fine particulate matter concentrations^23,31^. Due to the lack of actual landform data, the spatial distribution of PM_2.5_ was mainly simulated by interpolation. The future further study about satellite mapping of fine particulates should be based on geographically weighted regression.

### Effects of source emissions on spatial and temporal PM_2.5_ distributions

The sources of urban PM_2.5_ were complex, including exhaust of motor vehicles, emissions of power plants and industrial boilers, combustions of household coal and biomass, open waste incineration and the dusts^32–34^. Clarifying the contributions of main pollutant sources would be beneficial to the environmental managements and air pollution control measures.

Table 3 showed the detailed air pollutant emissions of Jiangsu from 2011 to 2015, that the emission of soot was increased by 127 thousand tons, while SO_2_ and NO_x_ reduced by 219 thousand and 468 thousand tons. Because NO_x_ and SO_2_ were the two key air pollutants for producing secondary PM_2.5_, they were of great significance to the decrease of PM_2.5_ pollution in this study area^35^. According to the structure of exhaust emissions, the percentage of industrial SO_2_ and NO_x_ emissions were decreased due to vigorously promoting clean energy and renovating coal fired boilers. In 2015, the total emissions of soot, NO_x_, and SO_2_ in Jiangsu province were 654, 1068, and 835 thousand tons, of which industrial emissions contributed 612, 754, and 795 thousand tons and accounted for 93.6%, 70.6%, and 95.2% of corresponding total emissions, respectively. In addition to industrial emissions, vehicle exhaust contributed significantly to NO_x_ emissions, accounting for 28.6%. As to the regional distribution, the anthropogenic emissions of soot, SO_2_ and NO_x_ at the heavy industrial city were higher than coastal city. To effectively control the urban air pollution, reductions of local coal, vehicular and industrial emissions, and the regional joint pollution prevention and control policies are necessary.

**Table 3:** Annual detail emission information of air pollution sources in Jiangsu from 2011 to 2015.

Sources of air pollutants	2011	2012	2013	2014	2015
**Soot emission (10000 tons)**	**52.74**	**44.32**	**50.00**	**76.38**	**65.45**
Industry	48.64	39.60	45.56	72.05	61.22
Urban life	1.20	1.85	1.71	1.82	1.94
Motor	2.88	2.85	2.70	2.48	2.27
Centralized treatment facilities	0.02	0.02	0.03	0.03	0.02
**SO**_**2**_ **emission (10000 tons)**	**105.4**	**99.20**	**94.18**	**90.48**	**83.51**
Industry	102.5	95.92	90.95	87.02	79.47
Urban life	2.85	3.25	3.20	3.43	4.03
Centralized treatment facilities	0.02	0.03	0.03	0.03	0.01
**NOx emissions (10000 tons)**	**153.6**	**145.0**	**133.8**	**123.2**	**106.8**
Industry	119.6	113.4	98.53	88.82	75.36
Urban life	0.62	0.65	0.61	0.64	0.86
Motor	33.35	33.90	34.62	33.74	30.50
Centralized treatment facilities	0.04	0.05	0.04	0.05	0.05

### Regional air pollutant transport

Besides local pollution sources, the typical monsoon climate plays an important role in the long distance transport of aerosols^18,36^. The typical life cycle of aerosols in the atmosphere was 3–10 days. To explore the impact of inter-regional air transport on pollutant concentrations, 72 h backward trajectory cluster and PSCF analysis were conducted for four typical regions in Jiangsu province.

**Figure 5:**
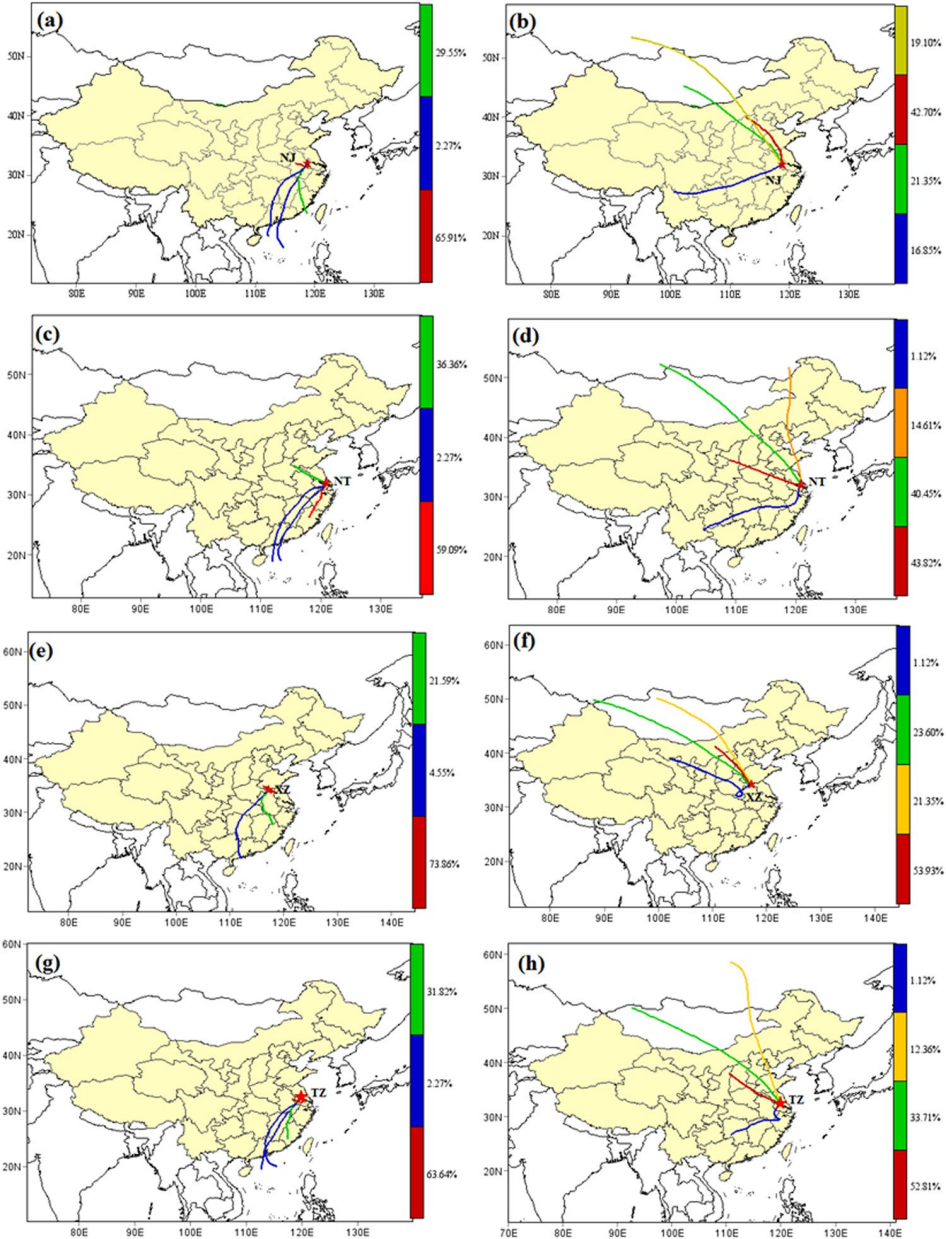
The 72-h backward trajectories clustering for four representative cities (XZ, northern heavy industrial city; NT, eastern coastal city; TZ, inland city; and NJ, developed city) during summer and winter.

Figure 5 compared the cluster analyses of them in summer and winter. Air pollution was found to be affected by the monsoon climate. In summer, the clustering percentage of air transport from nearby regions accounted for 65.9%, 59.1%, 73.9%, and 63.6%, respectively. Long-range air mass transport clusters account for a less proportion, and the directions were mainly from the southeast coast and the southern region. In winter, the air masses mainly come from the inland areas of the northwest, representing long-distance air mass transport, the transmission distance and vertical span were significantly larger than those in summer. During the process of winter air pollutants transmission, the study areas were impacted by the northwest polluted air masses originated from Inner Mongolia and along Shanxi, Hebei, Henan, Anhui and Shandong provinces^37^. The trajectory of the airflow was faster, indicated that the air mass rises to the free troposphere at the source, and moved to the downstream at a faster speed under the action of the wind speed. After reaching the research area, it entered the boundary layer after vertical mixing and sinking. In addition, pollution level was influenced by the monsoon climate and Siberia high pressure, the downdraft and stably stratified atmosphere help to increase PM_2.5_ pollution level in these cities^38,39^. These results of backward trajectory clustering analysis implied the significant effects of regional dispersion and meteorological conditions on regional air quality.

**Figure 6:**
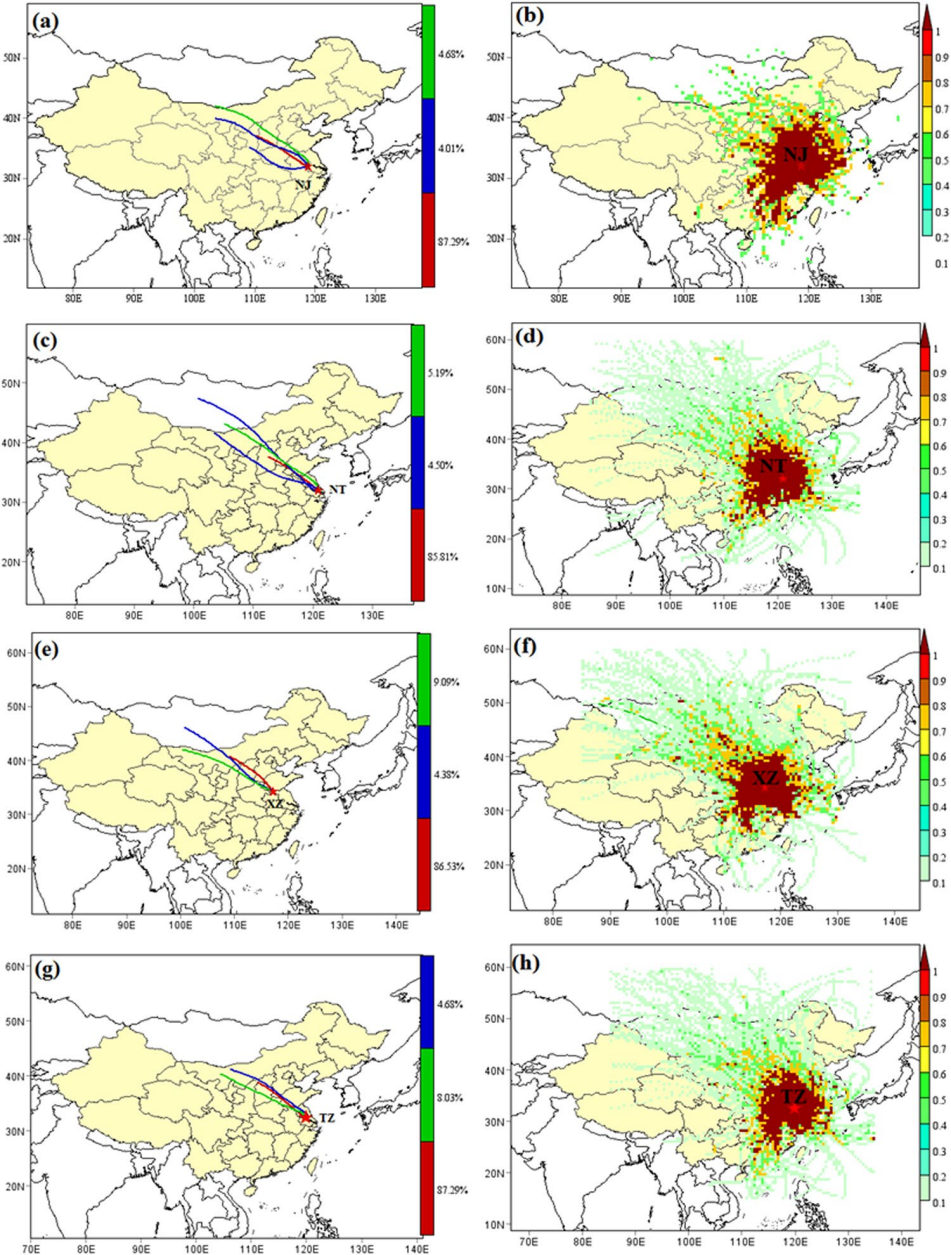
The 72-h backward trajectories clustering and PSCF for four representative cities (XZ, northern heavy industrial city; NT, eastern coastal city; TZ, inland city; and NJ, developed city) during 2017 based on PM_2.5_ concentration.

PSCF analysis was applied to explore the potential source region distribution of PM_2.5_. A PSCF analysis of PM_2.5_ combined with atmospheric data based on the backward trajectory for four representative cities in 2017 was showed in Fig. 6. The spatial patterns were similar, and the source areas with higher contribution rate were mainly distributed in neighboring provinces such as Anhui, Shandong, Zhejiang provinces, etc., indicating the significance of pollution in the nearby area^37^. In addition, due to the long-range air mass transport, the contribution values of Inner Mongolia, Hebei, Gansu, Ningxia, Guangdong and Fujian provinces reached 0.5–0.8, which increased the pollutant concentrations in the study area^40^. It should be noted that the PSCF analysis did not estimate the spatial distribution of all sources. The high potential contribution source area may coincide with the regional emission sources, but the low contribution value does not necessarily indicate low emissions in the area.

## Conclusions

Air PM_2.5_ pollution was still a significant environmental issue with varied spatial and temporal distributions. Typically in eastern China, 5-year data of 110 monitoring sites from all the 13 cities of a province were compared by statistics and illustrated by GIS. According to the intra-annual and inter-annual variations of PM_2.5_ in large-scale and long-term, the PM_2.5_ pollution contributed by industrial and traffic emissions was still obvious, but the level has reduced significantly in recent 5 years owing to the strengthened pollution control measures. PM_2.5_ concentrations were significantly higher in winter than summer, and were in an order of heavy industrial area > inland area > developed area > coastal area, due to different emission sources and meteorological conditions. Besides local primary emissions and secondary aerosol formation, the backward trajectory clustering analysis and potential source contribution factor (PSCF) analysis indicated that the typical monsoon climate played an important role in the aerosol transport. In summer, it was mainly affected by the updraft from near region, while in winter, the long-distance transport from the northwest had a significant impact on air pollution. Moreover, the nearby provinces were important potential pollution sources.

## Data and Methods

### Study areas

Jiangsu Province is an important part of the developed Yangtze River Delta located along the eastern coast of China (Fig. 4). It covers an area of 103 thousand km^2^ and has a population of 8.03 million. With Yellow Sea to its east, Jiangsu adjoins Anhui and Shandong provinces in the west and north respectively, with Zhejiang province and Shanghai city as neighbors in the southeast. It has a large area of plain as typical topography and dotted with two top-5 largest lakes in China. Situated in a transition belt from a subtropical to temperate zone, this province has a typical monsoon climate. Approximately demarcated by the Huai River, the south is subtropical monsoon climate and the north is warm moist monsoon climate. Generally, it is mild with moderate rainfall and four distinct seasons. Its economy is dominated by industrial activities with a GDP of 859 billion yuan (top 2 in China), and coal is the main energy source with an annual consumption of 258 million tons. Moreover, the number of motor vehicles reached 17.3 million in 2015, and 1232 polluting enterprises were monitored^41^. As an industry and transportation dense region, it was the highest PM_2.5_ emission province (0.28 million tons) in the year of 2014^42,43^. Associated with the economic developments, the problem of air pollution has become a key environmental issue that regulatory policies and pollution control measures were implemented and strengthened by recent years^44,45^. Therefore, it provided an ideal area to study the profound characteristics of air pollution in a large-space scale and long-term scale.

To characterize the air PM_2.5_ pollution patterns and mechanisms, this study analyzed air pollution and related factor data from all the 13 cities of Jiangsu Province, including Nanjing (NJ), Suzhou (SZ), Wuxi (WX), Changzhou (CZ), Nantong (NT), Xuzhou (XZ), and Lianyungang (LYG), Zhenjiang (ZJ), Huai’an (HA), Yancheng (YC), Taizhou (TZ), Suqian (SQ) and Yangzhou (YZ). Figure 4 showed the location of study area and air monitoring sites.

### Data sources

Based on the monitoring site information provided by the China Environmental Protection Monitoring Bureau, 110 national air quality monitoring stations in Jiangsu Province were used for spatial study (Table A2). Daily average of 24 h PM_2.5_ concentration and real-time concentration data (i.e., hourly average) from 18 January, 2013 to 31 December, 2017 were collected for intra-annual and inter-annual variation analyses. The meteorological data used for backward trajectory analysis was from the simultaneous global data assimilation system (GDAS) provided by the National Center for Environmental Prediction (NCEP) of USA, including temperature, relative humidity, surface precipitation, horizontal and vertical wind speeds. Data of pollution sources, emissions and economic development were obtained from the Statistical Yearbook of Jiangsu Province^41^. The numbers of companies with emissions were from the Self-Monitoring Information Release Platform of Focused Enterprises in Jiangsu Province (http://218.94.78.61:8080/newPub/web/home.htm).

### The GIS spatial analysis by Kriging interpolation method

The GIS is a comprehensive subject combining geography and cartography, remote sensing and computer science. GIS technology integrates the unique visual effect and geo-analysis function of maps with general database operations^46^, and has been widely applied in environmental science^47–49^. Interpolation method is an important content of spatial statistical analysis in GIS. Among the various interpolation methods, the Kriging interpolation is flexible and can fully utilize the data exploratory analysis tools to improve the efficiency of spatial analysis effectively^20^. It can use the statistical characteristics of known samples to quantify the spatial autocorrelation between measurement points, highlighting the overall distribution trend, increasing the data fidelity, and has the highest prediction accuracy for normal data^50^. In this study, the Kriging interpolation method was applied to explore the spatial distribution of PM_2.5_ concentration data of 110 stations during 2013 and 2017, thus reveal the PM_2.5_ pollution patterns in the overall study area intuitively.

### Long-range air mass transportation by trajectory calculation

As a long-range source analysis technique based on observational difference or simulated meteorological field^51,52^, the 72-h backward air trajectories clustering and PSCF analysis were calculated using the HYSPLIT model (http://ready.arl.noaa.gov/HYSPLIT.php). The meteorological data as 1° × 1°GDAS data, and the trajectory calculation points were NJ (Nanjing, 32.06N, 118.78E, 1000 m), NT (Nantong, 31.99N, 120.88E, 1000 m), XZ (Xuzhou, 34.25N, 117.21E, 1000 m) and TZ (Taizhou, 32.49N, 119.90E, 1000 m) were used in the trajectory calculation, and two trajectory were calculated every day at 0:00 and 12:00 (UTC), starting at: 2017.1.1 0:00 to 2018.1.1 0:00.

PSCF was a trajectory-based gridded statistical analysis method that can obtain the spatial distribution of pollution sources in a semi-quantitative manner^40^. The basic concept of PSCF was to combine the air mass trajectory with the atmospheric component data to generate a conditional probability in a given region which was divided into i*j small grids. The conditional probability was combined with the air mass trajectory to describe the possible spatial distribution of geographic source locations. The PSCF_ij_ value of the ij grid point was

$$PSC{F}_{ij=\frac{{m}_{ij}}{{n}_{ij}}=\frac{thenumberoftrajectoryendpoints{\rm{reaching}}{\rm{the}}{\rm{pollution}}{\rm{threshold}}{\rm{in}}{\rm{the}}{\rm{ij}}{\rm{grid}}{\rm{points}}}{thenumberofendpointsintheijgridpoints}}$$

In order to reduce the uncertainty caused by the small n_ij_ value, the weight function w_ij_ was introduced, and when the number of trajectory endpoints in the ij grid points was less than about three times the average number of trajectory endpoints, w_ij_ decreased the PSCF_ij_ value. The PSCF method was widely used in atmospheric chemistry research, such as analysis of particulate matter in the atmosphere and potential source distribution of inorganic components in the particles. It was generally considered that when PSCF ≤ 0.5, it was a low contribution source region, and conversely, a high contribution source region. The area analyzed in this paper was located in 85–135°E, 15–60°N, and the grid resolution was 0.5° × 0.5°, Weight function

$${\rm{wij}}=\{\begin{array}{cc}1.00\, & 12 < {n}_{ij}\\ 0.70\, & 6 < {n}_{ij}\le 12\\ 0.42\, & 4 < {n}_{ij}\le \,6\\ 0.17\, & {n}_{ij}\le 4,\end{array}$$

the pollution threshold was set to the primary standard of PM_2.5_ concentration (35 μg.m^−3^).

## Supplementary information

Supplementary
