# Supplementary material for: Spatio-temporal variations and factors of a provincial PM2.5 pollution in eastern China during 2013–2017 by geostatistics
Source: Sci Rep. 2019 Mar 5;9:3613. doi: 10.1038/s41598-019-40426-8 (PMC6401087; doi:10.1038/s41598-019-40426-8)
Supplement: Supplementary file 1 — Supplementary [file 41598_2019_40426_MOESM1_ESM.docx]

**Scientific Reports**

**Supplementary Materials**

**Spatio-temporal variations and factors of a provincial PM**_2.5_ **pollution in eastern China during 2013-2017 by geostatistics**

**Xue Sun, Xiao-San Luo^*^, Jiangbing Xu, Zhen Zhao, Yan Chen, Lichun Wu, Qi Chen, Dan Zhang**

*International Center for Ecology, Meteorology, and Environment, Collaborative Innovation Center of Atmospheric Environment and Equipment Technology (AEET), School of Applied Meteorology, Nanjing University of Information Science & Technology, Nanjing 210044, China*

(*Corresponding Author, Email: [xsluo@nuist.edu.cn](mailto:xsluo@nuist.edu.cn), Tel: +86-25-58731294, https://orcid.org/0000-0003-4314-7216)

**Captions of figures and tables**

**Table A1** Information of the 110 air monitoring sites scattering in Jiangsu province, China

**Table A2** Concentration limits of guidelines for environmental air pollutants in China

**Fig. A1** Average annual concentrations of air PM_2.5_ in 13 cities of Jiangsu, China during 2013-2017

**Fig. A2** The proportions of days with different PM_2.5_ levels during 2013-2017

**Table A1** Information of the 110 air monitoring sites scattering in Jiangsu province, China

| Sites | Longitude(E) | Latitude（N） | Sites | Longitude(E) | Latitude（N） |
| --- | --- | --- | --- | --- | --- |
| NJ1 | 118.80 | 32.11 | SZ12 | 120.53 | 31.87 |
| NJ2 | 118.75 | 32.06 | SZ13 | 120.55 | 31.88 |
| NJ3 | 118.78 | 32.07 | SZ14 | 120.95 | 31.39 |
| NJ4 | 118.78 | 32.01 | SZ15 | 121.00 | 31.38 |
| NJ5 | 118.80 | 32.03 | SZ16 | 120.64 | 31.16 |
| NJ6 | 118.80 | 32.08 | SZ17 | 120.64 | 31.17 |
| NJ7 | 118.63 | 32.09 | SZ18 | 120.66 | 31.17 |
| NJ8 | 118.74 | 32.01 | SZ19 | 121.12 | 31.45 |
| NJ9 | 118.91 | 32.11 | SZ20 | 121.14 | 31.42 |
| WX1 | 120.27 | 31.49 | NT1 | 120.91 | 31.96 |
| WX2 | 120.28 | 31.62 | NT2 | 120.86 | 32.00 |
| WX3 | 120.29 | 31.56 | NT3 | 120.87 | 32.02 |
| WX4 | 120.24 | 31.50 | NT4 | 120.94 | 31.93 |
| WX5 | 120.35 | 31.58 | NT5 | 120.81 | 32.04 |
| WX6 | 120.35 | 31.55 | NT6 | 121.07 | 32.10 |
| WX7 | 120.25 | 31.56 | NT7 | 121.17 | 31.90 |
| WX8 | 120.29 | 31.68 | NT8 | 121.15 | 31.90 |
| WX9 | 120.27 | 31.92 | LYG1 | 119.18 | 34.59 |
| WX10 | 120.24 | 31.91 | LYG2 | 119.36 | 34.70 |
| WX11 | 120.30 | 31.92 | LYG3 | 119.36 | 34.75 |
| WX12 | 119.82 | 31.35 | LYG4 | 119.15 | 34.59 |
| WX13 | 119.79 | 31.37 | LYG5 | 119.37 | 34.75 |
| XZ1 | 117.17 | 34.28 | LYG6 | 119.35 | 34.70 |
| XZ2 | 117.19 | 34.24 | LYG7 | 119.14 | 34.59 |
| XZ3 | 117.26 | 34.22 | HA1 | 119.04 | 33.60 |
| XZ4 | 117.24 | 34.29 | HA2 | 119.01 | 33.58 |
| XZ5 | 117.29 | 34.28 | HA3 | 118.99 | 33.61 |
| XZ6 | 117.18 | 34.29 | HA4 | 119.14 | 33.50 |
| XZ7 | 117.18 | 34.29 | HA5 | 119.01 | 33.63 |
| XZ8 | 117.17 | 34.18 | YC1 | 120.12 | 33.40 |
| XZ9 | 117.12 | 34.47 | YC2 | 120.16 | 33.39 |
| CZ1 | 120.00 | 31.76 | YC3 | 120.23 | 33.39 |
| CZ2 | 119.94 | 31.70 | YC4 | 120.16 | 33.37 |
| CZ3 | 119.91 | 31.91 | YC5 | 120.13 | 33.37 |
| CZ4 | 119.97 | 31.81 | YZ1 | 119.46 | 32.39 |
| CZ5 | 120.05 | 31.77 | YZ2 | 119.40 | 32.41 |
| CZ6 | 119.89 | 31.79 | YZ3 | 119.39 | 32.38 |
| CZ7 | 120.03 | 31.76 | YZ4 | 119.46 | 32.41 |
| CZ8 | 119.95 | 31.78 | YZ5 | 119.56 | 32.47 |
| CZ9 | 119.96 | 31.81 | ZJ1 | 119.68 | 32.19 |
| CZ10 | 119.46 | 31.40 | ZJ2 | 119.49 | 32.22 |
| CZ11 | 119.44 | 31.32 | ZJ3 | 119.43 | 32.13 |
| CZ12 | 119.58 | 31.75 | ZJ4 | 119.44 | 32.19 |
| CZ13 | 119.58 | 31.74 | ZJ5 | 119.15 | 31.96 |
| SZ1 | 120.56 | 31.25 | ZJ6 | 119.18 | 31.96 |
| SZ2 | 120.63 | 31.29 | TZ1 | 119.90 | 32.49 |
| SZ3 | 120.59 | 31.30 | TZ2 | 119.92 | 32.46 |
| SZ4 | 120.60 | 31.33 | TZ3 | 119.89 | 32.46 |
| SZ5 | 120.61 | 31.27 | TZ4 | 119.88 | 32.33 |
| SZ6 | 120.54 | 31.30 | TZ5 | 119.88 | 32.32 |
| SZ7 | 120.67 | 31.31 | SQ1 | 118.29 | 33.95 |
| SZ8 | 120.64 | 31.37 | SQ2 | 118.24 | 33.96 |
| SZ9 | 120.76 | 31.67 | SQ3 | 118.32 | 33.95 |
| SZ10 | 120.72 | 31.67 | SQ4 | 118.28 | 33.97 |
| SZ11 | 120.73 | 31.66 | SQ5 | 118.27 | 33.96 |

| Pollutants | Average time | Concentration limit | | Unit |
| --- | --- | --- | --- | --- |
|  |  | Standards-GradeⅠ | Standards-GradeⅡ |  |
| SO_2_ | The annual average | 20 | 60 | μg/m^3^ |
|  | 24-hour average | 50 | 150 |  |
|  | 1 hour average | 150 | 500 |  |
| NO_2_ | The annual average | 40 | 40 |  |
|  | 24-hour average | 80 | 80 |  |
|  | 1 hour average | 200 | 200 |  |
| CO | 24-hour average | 4 | 4 | mg/m^3^ |
|  | 1 hour average | 10 | 10 |  |
| O_3_ | Maximum 8 hours daily | 100 | 160 | μg/m^3^ |
|  | 1 hour average | 160 | 200 |  |
| PM_10_ | The annual average | 40 | 70 |  |
|  | 24-hour average | 50 | 150 |  |
| PM_2.5_ | The annual average | 15 | 35 |  |
|  | 24-hour average | 35 | 75 |  |

**Table A2** Concentration limits of guidelines for environmental air pollutants in China


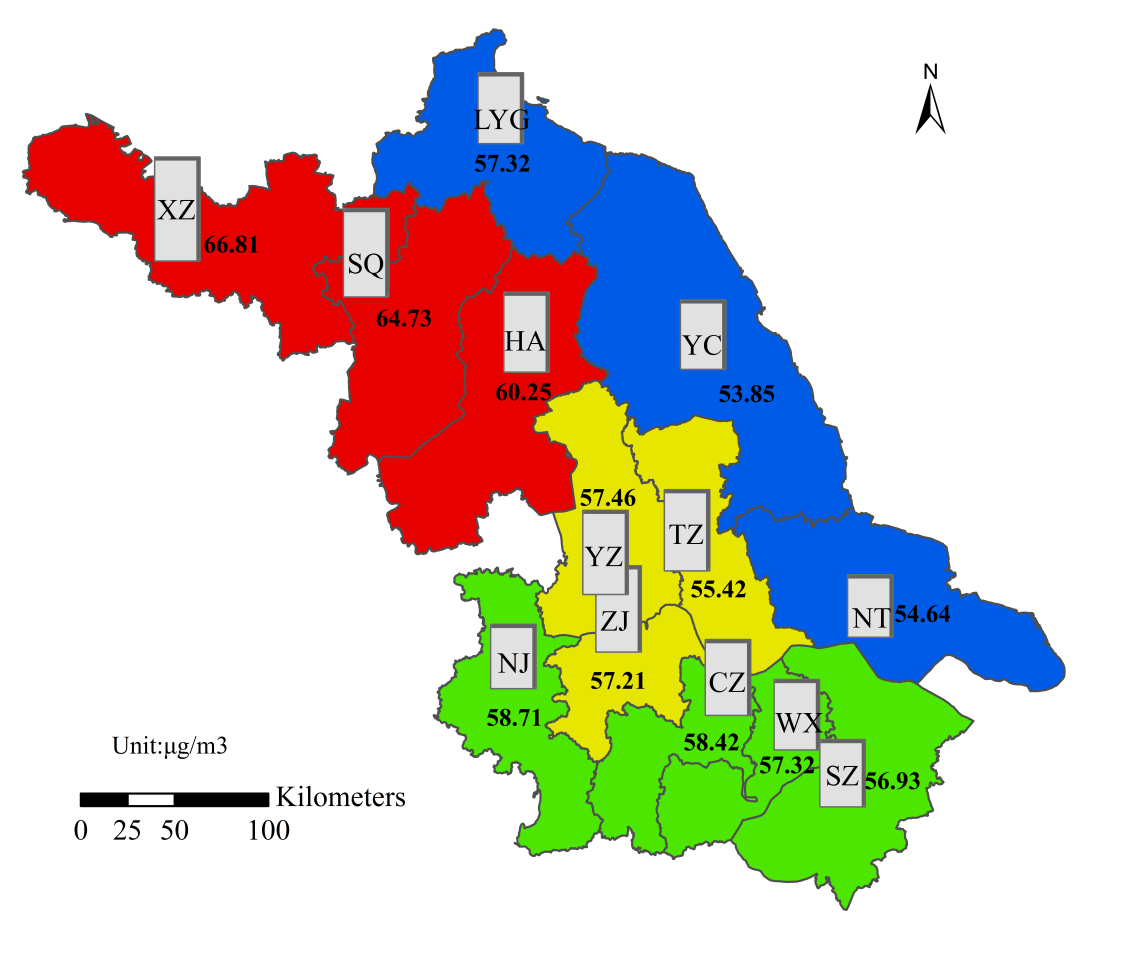


**Fig. A1** Average annual concentrations of air PM_2.5_ in 13 cities of Jiangsu, China during 2013- 2017


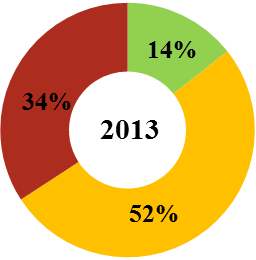

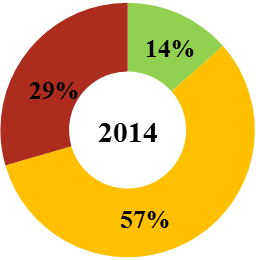

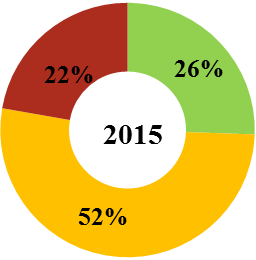

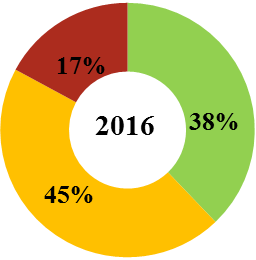

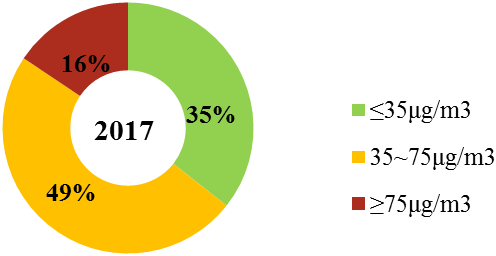


(a)

(b)

(c)

(d)

(e)

**Fig. A2** The proportions of days unnder different PM_2.5_ levels during 2013-2017
